# Topical Corticosteroid Phobia in Childhood Atopic Dermatitis: A Study Using the TOPICOP Score

**DOI:** 10.1155/drp/7996688

**Published:** 2026-03-06

**Authors:** Mateja Starbek Zorko, Vid Bukovec, Tanja Fantulin

**Affiliations:** ^1^ Department of Dermatology and Veneric Diseases, Faculty of Medicine, University of Ljubljana, Ljubljana, Slovenia, uni-lj.si; ^2^ Department of Dermatovenereology, University Medical Centre Ljubljana, Ljubljana, Slovenia, kclj.si; ^3^ Emergency Department, Division of Internal Medicine, University Medical Centre Ljubljana, Ljubljana, Slovenia, kclj.si; ^4^ Department of Dermatovenereology, Faculty of Medicine, University of Maribor, Maribor, Slovenia, um.si

**Keywords:** atopic dermatitis, child, corticosteroids, cross-sectional studies, parents, quality of life, severity of illness index, topical

## Abstract

**Background:**

Topical corticosteroid phobia (CSP) is a significant barrier to the effective treatment of atopic dermatitis (AD). This is the first study to evaluate the CSP prevalence among parents of children with AD in Slovenia and to identify contributing factors.

**Methods:**

We conducted a cross‐sectional study between March 2021 and December 2023. Parents of children with dermatologist‐confirmed AD, aged 3 months to 18 years, completed the validated TOPICOP questionnaire, supplemental questions and the (F)DLQI questionnaire. The SCORAD index was used to assess the severity of their children’s AD disease. Statistical analyses included the Shapiro–Wilk test, two‐tailed independent *t*‐test, ANOVA, followed by Tukey post hoc testing and the Pearson correlation coefficient.

**Results:**

Among 117 parents (81.2% mothers), the mean TOPICOP score was 48.2% (SD 15.1). Fear was the highest‐scoring TOPICOP domain (53.7%). CSP was significantly higher in parents of children with more severe AD based on SCORAD (*p* = 0.033) and in families with higher (F)DLQI scores (*r* = 0.311, *p* = 0.002). Notably, our results suggest that parents of children in single‐parent households had significantly higher CSP (*p* = 0.035), a novel finding that warrants cautious interpretation due to the small subgroup size. Information obtained online about the potential dangers of topical corticosteroids (TCS) correlated with higher CSP (*p* < 0.001).

**Conclusion:**

CSP is prevalent among Slovenian parents of children with AD and is particularly pronounced in cases of more severe disease and single‐parent households, a novel and previously undescribed finding. Given that CSP is often influenced by nonmedical information sources, structured education and support by all healthcare providers is essential.

## 1. Introduction

Atopic dermatitis (AD) is one of the most common chronic skin conditions globally, affecting both children and adults [1]. The prevalence varies significantly across geographic regions, with higher rates reported in developed countries and urban areas [2, 3]. According to the World Health Organization, AD affects approximately 15%–20% of children and 1%–3% of adults [1, 4], although many recent studies show that AD can affect up to 10% of the adult population [5, 6]. The disease is chronic and often relapsing, characterised by intense itching and discomfort that profoundly impacts patients’ quality of life [7]. These challenges can lead to sleep disturbances, diminished self‐esteem and diminished performance in school or at work [8].

Management of AD requires a comprehensive and individualised approach, with emphasis on symptom control and quality‐of‐life improvement. Key strategies include trigger avoidance, regular skincare, topical therapies, phototherapy and systemic treatments in severe cases [9]. Topical corticosteroids (TCS) and topical calcineurin inhibitors are first‐line treatments for flares, with TCS often favoured due to their efficacy and minimal side effects when used appropriately [10]. However, the chronic nature of AD necessitates prolonged and repeated TCS use, which can raise concerns about adverse effects, including skin atrophy, stretch marks and telangiectasia. Proper usage significantly minimises these risks, and TCS have been demonstrated to be safe for long‐term application [11]. The risk of ‘corticosteroid dependence’ and ‘withdrawal syndrome’ after discontinuation of TCS is small and overestimated when used correctly [12].

Education is a cornerstone of AD management. Patient and caregiver education on preventive measures, proper skincare and the safe use of medications is critical to improving adherence and outcomes. However, topical corticosteroid phobia (CSP), encompassing fear and hesitation regarding corticosteroid use, poses a significant barrier to effective AD treatment [13]. CSP is prevalent among patients, caregivers and even healthcare professionals, with reported rates ranging from 21.0% to 83.7%, depending on the country and the definition used [14]. CSP is more common in women, individuals with lower educational levels and those with limited health literacy, often resulting in delayed or inadequate treatment of AD flares [14–17]. Conversely, adequate education from dermatologists reduces CSP prevalence and improves treatment adherence [18].

Understanding and addressing the causes of CSP is vital for optimising AD management. Dermatologists, paediatricians and other healthcare providers, such as pharmacists, play a key role in identifying CSP and offering targeted education and support to ensure the safe and effective use of corticosteroids [19]. This approach not only alleviates fear but also enhances treatment outcomes and patient quality of life [20]. This study aimed to determine the prevalence of CSP among Slovenian parents of children with AD and identify associated risk factors.

## 2. Methods

### 2.1. Study Design and Population

This cross‐sectional observational study was conducted at the Paediatric Unit of the Department of Dermatovenereology, University Medical Centre Ljubljana. Data collection took place between 5 March 2021, and 30 December 2023. The study included parents of children with a confirmed diagnosis of AD who met the inclusion and exclusion criteria and agreed to participate. All participants were informed about the study’s purpose and asked to complete a predesigned questionnaire developed by experienced dermatological experts. The consistency and accuracy of the parents’ responses were relied upon throughout the data collection process.

The inclusion criteria required the parents’ written consent to participate, a confirmed diagnosis of AD in a child aged between 3 months and 18 years and prior use of TCS for the treatment of AD in the child. The exclusion criteria disqualified parents whose children were younger than 3 months or older than 18 years, those whose children had another widespread dermatosis and those who did not provide complete consent. Additionally, families whose children had not been treated with TCS for AD or were undergoing systemic therapy that could influence the disease’s clinical presentation were excluded. The diagnosis of AD in all children who were included was confirmed by experienced dermatologists based on the clinical presentation and typical disease course.

### 2.2. Data Collection

To assess CSP, the study utilised the translated Topical Corticosteroid Phobia (TOPICOP) questionnaire [21], which was already used in previous studies of CSP in Slovenia [22]. Scores are expressed as a percentage, with higher scores indicating greater CSP.

Supplementary data included demographics, AD duration, a family history of skin disease, treatment history and sources of received warnings about the dangers of TCS use. Sources of warnings about the potential hazards of TCS use were assessed using a multiple‐choice question, where parents could select all applicable sources (e.g., Internet, friends and family, personal physicians, dermatologist or pharmacists).

The clinical severity of AD was assessed by an experienced dermatologist using the Scoring Atopic Dermatitis (SCORAD) index [9, 23]. The final score ranges from 0 to 103, categorising AD as mild (SCORAD < 25), moderate (SCORAD 25–50) or severe (SCORAD > 50) [9, 23].

In addition to SCORAD, the impact of AD on quality of life was assessed using the translated Dermatology Life Quality Index (DLQI) [24]. This questionnaire aims to evaluate the effects of skin disease on various aspects of life, including symptoms, well‐being, daily activities, leisure, work or school, personal relationships and treatment. Scores range from 0 to 30, with higher scores reflecting a greater impact on quality of life. For children younger than 4 years, the translated Family Dermatology Life Quality Index (FDLQI) was used as an alternative. This tool mirrors the DLQI in structure and scoring but focuses on the impact of AD on the entire family, with responses provided by the parents [25].

### 2.3. Statistical Methods

All collected questionnaires were included in the analysis, and the missing items were not imputed. Out of 117 participants, 94 provided sufficient data for the TOPICOP score calculation. Missing items within the TOPICOP questionnaire were excluded, and the total score was calculated as a percentage of the items answered. This method was also applied to the individual TOPICOP domains (knowledge and beliefs, fears and TCS use). To ensure a comprehensive assessment of the impact on quality of life, the DLQI and FDLQI scores were combined and analysed collectively as (F)DLQI.

The Shapiro–Wilk test was employed to evaluate the normal distribution of the data. For normally distributed data, a two‐tailed independent *t*‐test was used to compare groups based on the child’s sex, social status and observed side effects. To compare multiple groups, such as sources of information on TCS risks, a one‐way analysis of variance (ANOVA) was performed, followed by Tukey post hoc testing to analyse differences between subgroups. Pearson correlation coefficients were calculated to examine the relationships between TOPICOP scores and (F)DLQI scores, as well as between TOPICOP scores and the duration of AD symptoms.

Statistical analyses were conducted using IBM SPSS Statistics Version 29.0 (IBM, United States). A *p* value of < 0.05 was considered statistically significant.

## 3. Results

### 3.1. Demographics

A total of 117 parents of children diagnosed with AD, aged between 4 months and 18 years, participated in the study. The children were patients at the Paediatric Department of the Dermatology Clinic, University Medical Centre Ljubljana. Demographic data of the children and their parents are summarised in Table 1.

**TABLE 1:** Demographics of patients and their parents included in the study.

*Sex of the parent*	*n* ^†^ = 117
M^‡^, *n* (%)	22 (18.8)
F^§^, *n* (%)	95 (81.2)

*Sex of the child*	*n* = 117
M, *n* (%)	57 (48.7)
F, *n* (%)	60 (51.3)

*Children’s age in years*	*n* = 117
Mean (SD^¶^)	5.9 (4.3)

*Duration of* *A* *D* ^⸸^ *in years*	*n* = 85
Mean (SD)	5.1 (3.9)

*Lives with parents*	*n* = 115
One of them, *n* (%)	11 (9.6)
Both, *n* (%)	104 (90.4)

^†^Number of answers.

^‡^Male.

^§^Female.

^¶^Standard deviation.

^⸸^Atopic dermatitis.

### 3.2. Corticosteroid Phobia Prevalence

The results of the TOPICOP questionnaire were normally distributed. The mean total TOPICOP index was 48.2% (SD 15.1, *n* = 94). Among the individual domains, the highest scores were observed in the domain of fears (mean 53.7%, SD 25.2), while the lowest were in the domain of TCS use (mean 44.2%, SD 24.7). Detailed averages for all domains are presented in Table 2.

**TABLE 2:** Average TOPICOP index by domain.

	Mean (%)	SD^¶^ (%)	*n* ^†^
TOPICOP total	48.2	15.1	94
TOPICOP knowledge and beliefs	48.4	14.9	93
TOPICOP fears	53.7	25.2	94
TOPICOP use	44.2	24.7	94

^¶^Standard deviation.

^†^Number of answers.

### 3.3. Risk Factors for CSP

**FIGURE 1:**
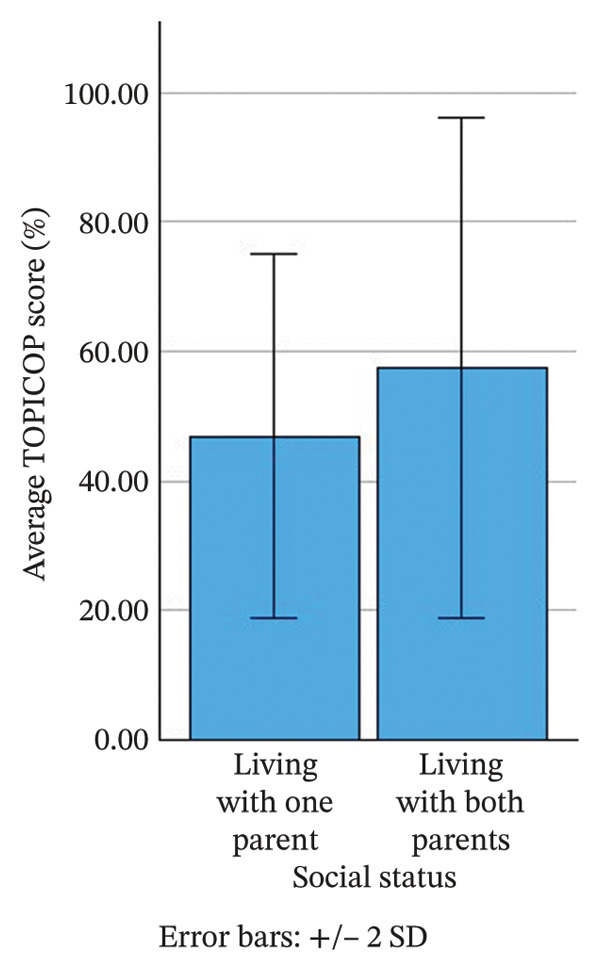
Comparison of the TOPICOP index between children from single‐ and two‐parent households.

Children were more than four times as likely to be accompanied by their mothers compared to their fathers. However, no statistically significant difference in the total TOPICOP index was observed between groups based on the accompanying parent (*p* = 0.901). Additionally, the correlation between the total TOPICOP index and the duration of AD symptoms was not statistically significant (*p* = 0.362). More than 90% of the children, however, lived with both parents, while the remainder lived with one parent. The mean TOPICOP index was 10.6% higher in children who did not live with both parents (*p* = 0.035), as shown in Figure 1. While this difference was statistically significant, the findings should be interpreted with caution due to the small size of the single‐parent subgroup (*n* = 11).

### 3.4. AD Severity

Based on SCORAD assessments, the severity of AD was distributed approximately evenly among mild (33.0%), moderate (36.5%) and severe (30.4%) categories. Interestingly, parents often rated their children’s AD severity higher than dermatologists. Nearly half of the parents (47.4%) subjectively assessed their child’s AD as severe.

**FIGURE 2:**
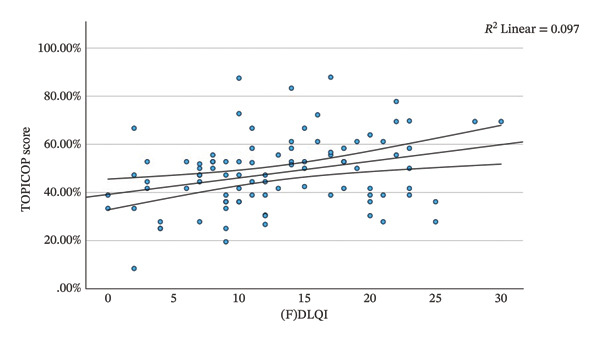
Correlation between the total TOPICOP index and the (F)DLQI.

TOPICOP scores in relation to AD severity were as follows:

• In the moderate SCORAD group, mean TOPICOP was 8.9% higher than in the mild group (*p* = 0.033), with an 18.3% higher score in the fear domain (*p* = 0.005).
• Parents of children with mild AD (as assessed by the parents) demonstrated a 13.6% lower mean TOPICOP score in the knowledge and beliefs domain compared to parents of children with severe AD (*p* = 0.012).
• The mean (F)DLQI score was 14.0 (SD 6.8, *n* = 116). A higher (F)DLQI index was positively correlated with a higher total TOPICOP index (*r* = 0.311, *p* = 0.002). The correlation is shown in Figure 2.
• The differences observed in the remaining groups did not reach statistical significance.

### 3.5. Perceived Side Effects

Nearly all (96.4%) of the 84 parents who responded to this question reported improvements in their child’s condition with TCS use. However, 25.3% of 83 parents also observed side effects. The most reported side effects were redness (6.0%), depigmentation (4.8%) and worsening of the disease after discontinuation of TCS use compared to its severity before treatment (4.8%). Other reported side effects included itching, rash and various less common reactions. Parents who observed side effects had a mean TOPICOP score 7.3% higher than those who did not, although this difference did not reach statistical significance (*p* = 0.067).

### 3.6. Sources of Information on TCS Dangers

**FIGURE 3:**
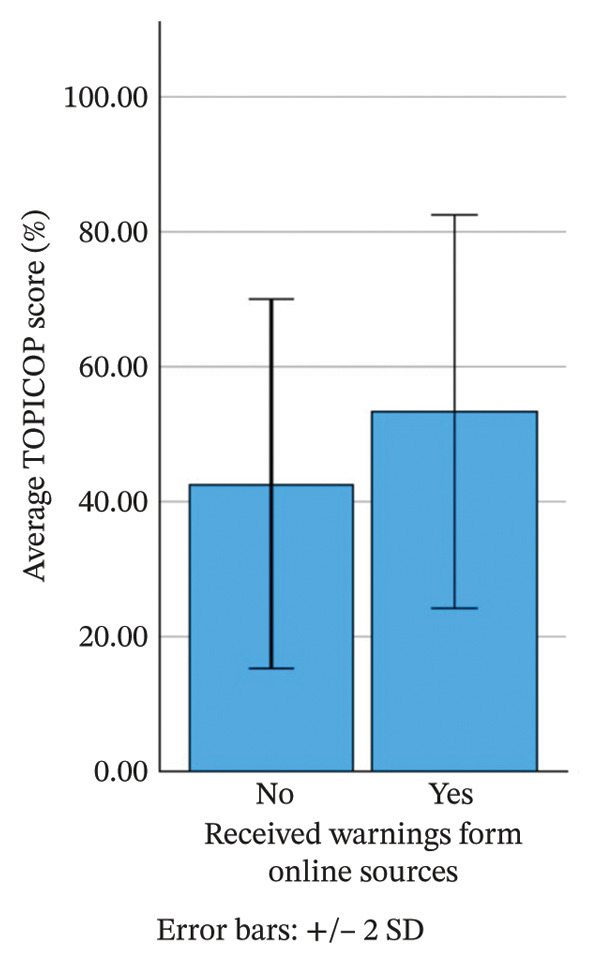
Comparison of the TOPICOP index between parents who received information online and those who did not.

Only 7 (6.0%) parents stated that they had not been informed of potential dangers associated with TCS use. Sources of information on the potential hazard of TCS are shown in Table 3. Parents who received information about the potential dangers of TCS use online had a 10.7% higher mean TOPICOP index compared to those who did not (*p* < 0.001), as shown in Figure 3. Conversely, parents who did not receive warnings about the dangers of TCS from any source had a lower mean TOPICOP index by 18.7% (*p* = 0.014). No significant differences were observed for other warning sources, such as dermatologists, friends, family members, personal physicians or pharmacists.

**TABLE 3:** Sources of corticosteroid hazard warnings.

	*n* ^†^ = 116
Internet, *n* (%)	64 (55.2)
A friend, acquaintance or family member, *n* (%)	55 (47.4)
Personal physician, *n* (%)	44 (37.9)
Pharmacist, *n* (%)	42 (36.2)
Dermatologist, *n* (%)	37 (31.9)
Other sources, *n* (%)	5 (4.3)

^†^number of answers.

Notably, 36 (31.9%) parents felt they had not received enough information about TCS use from their dermatologist. While this group had a higher average TOPICOP index by 1.7% than those who felt sufficiently informed, the difference was not statistically significant (*p* = 0.636).

## 4. Discussion

This is the first study on the origin and prevalence of CSP among parents of children with AD in Slovenia. Given the distinct features of Slovenia’s healthcare system, which includes universal health coverage and potential cultural attitudes toward medication use, these findings provide valuable context for understanding CSP in a Central European setting.

### 4.1. Corticosteroid Phobia

The mean TOPICOP index was 48.2%, which is comparable to previous studies from other countries using the TOPICOP index [26, 27]. This supports our hypothesis that CSP is present and prevalent in Slovenia among parents of children with AD. The prevalence underscores CSP’s negative impact on adherence to TCS treatment [28–30]. The prevalence of CSP among parents of children with AD is also comparable to that observed in adult Slovenian patients with AD or psoriasis, where the mean total TOPICOP index was 44.0% [22].

It is important to note the discrepancy between the total number of participants (*n* = 117) and those who completed the TOPICOP questionnaire (*n* = 94). This could potentially introduce a nonresponse bias, as parents with more pronounced views on TCS might have been more likely to complete the survey.

Contrary to our expectations and previous studies [22, 26], we noted no statistically significant differences in the overall TOPICOP index based on the sex of the parent. However, our study suggests that social status may be a significant risk factor for CSP. Children living with a single parent had significantly higher CSP levels, despite the small sample size. To our knowledge, this is the first study suggesting a relationship between single parenthood and CSP of the parent of a child with AD. While this finding is novel, it must be interpreted with caution as other confounding factors, such as socioeconomic stress, lower household income or differences in health literacy, which were not fully controlled for in our study, might also contribute to this observation. This nevertheless indicates a need to pay extra attention to children in single‐parent households and ensure that all caregivers are educated about the role of TCS in AD treatment.

### 4.2. AD Severity

Parents often perceived their child’s AD as more severe than indicated by SCORAD. This discrepancy may stem from parents basing their assessment on cumulative experiences of AD flare‐ups, while SCORAD provides a single‐visit evaluation. CSP was higher in more severe forms of AD, supporting our hypothesis and findings from previous studies [16, 31]. Both the total TOPICOP index and the fear domain index were higher in the moderate group compared to the mild group (as assessed by SCORAD). Additionally, the knowledge and beliefs domain scores were higher in the parent‐assessed severe group compared to the mild group. The lack of statistical significance in some categories is likely due to the study’s limited statistical power, highlighting the need for further research with larger samples.

The association between CSP and AD severity was also confirmed by the correlation between higher (F)DLQI scores and higher total TOPICOP indices. The mean (F)DLQI in our study was 14.0, which is higher than the mean DLQI of 10.7 reported in a similar study from Italy, where a statistically significant relationship between DLQI and TOPICOP was also demonstrated [32].

Interestingly, our study shows that CSP was higher in more severe forms of AD regardless of how severity was determined (parental assessment, (F)DLQI or SCORAD). Given the cross‐sectional nature of our study, the relationship between CSP and AD severity is likely bidirectional. While severe AD may increase parental fear due to more frequent TCS exposure, high CSP can conversely lead to treatment nonadherence, resulting in poorer disease control and increased clinical severity. Therefore, it is unclear whether parents are more fearful of corticosteroids because severe AD requires more frequent use of TCS or whether insufficient TCS use due to CSP exacerbates the disease course.

### 4.3. Sources of Information on TCS Dangers

Parents of children with AD were more likely to learn about the dangers of corticosteroids from the Internet than from dermatologists, friends, family members, personal physicians or pharmacists. Previous studies have shown, however, that pharmacists and general practitioners, on average, have higher TOPICOP indices than dermatologists and may inadvertently contribute to CSP [33]. Negative warnings from pharmacists have also been identified as a significant barrier to successful treatment [30].

Our study confirmed that parents who obtained information about TCS dangers online had significantly higher CSP levels, consistent with prior research. Information found on the Internet is often unreliable or incorrect. It often propagates exaggerated risks of TCS side effects and promotes alternative treatments [34], contributing to CSP [31].

Our findings highlight the need for dermatologists to provide accurate, evidence‐based information to patients and parents, enabling them to critically evaluate information from other sources. Nearly a third of parents felt they did not receive enough information from dermatologists about TCS. Surprisingly, there were no significant differences in CSP rates between the groups, likely due to the small sample size and limited statistical power.

### 4.4. Advantages and Limitations of the Study

The primary strength of this study is the use of the TOPICOP questionnaire, which is an internationally approved tool for assessing CSP [35]. Its use allows for comparisons with existing and future studies. Additionally, the SCORAD index and the (F)DLQI questionnaires further assessed AD severity, aligning with treatment guidelines for various dermatoses [24] and making our findings comparable internationally.

However, the study’s main limitation is its small sample size, resulting in limited statistical power. The study was also conducted at a single tertiary healthcare centre, making the findings less representative of the general population, particularly of those with milder forms of AD, who are often managed by family medicine specialists or paediatricians.

## 5. Conclusions

Our study emphasises the importance of providing accurate and reliable information to patients and their parents to ensure adherence to AD treatment with TCS. Our research confirms the need for further studies on the causes of CSP to better understand AD patients and their parents. Addressing their fears and doubts about TCS use more effectively can improve adherence to AD treatment. Discovering the causes of CSP also provides opportunities for targeted interventions; therefore, it is crucial for dermatologists, paediatricians and other healthcare providers to actively identify CSP in patients or parents of children with AD and provide appropriate education and support [19]. Multidisciplinary programs and video‐based education for parents of children with AD have already proven successful abroad [20, 36] and could be beneficial in Slovenia as well.

## Funding

The research was approved and funded within the research project of the University Medical Centre Ljubljana, grant number TP 20200183, entitled ‘Corticosteroid phobia among dermatological patients with chronic inflammatory dermatoses’. It lasted 3 years (2020–2023), and the project leader was Assist. Prof. Mateja Starbek Zorko, MD, PhD. The funding source was not involved in the study design, data collection, analysis or interpretation of the results.

## Ethics Statement

The research received approval on 4 March 2021, from the Medical Ethics Commission of the Republic of Slovenia under assessment number 0120‐454/2020/6. All participants received detailed verbal and written instructions about the purpose of the research, after which they were asked to sign a consent form to participate in the study voluntarily. The principles of the Declaration of Helsinki were considered in our research. Participants were not compensated for their participation.

## Conflicts of Interest

This study was funded by the University Medical Centre Ljubljana. Two authors are employed at this institution, which could be perceived as a potential conflict of interest. However, the funding body had no role in the conception, design, conduct, analysis or reporting of the study. The other author declares no conflicts of interest.

## Data Availability

The data that support the findings of this study are available upon request from the corresponding author. The data are not publicly available due to privacy or ethical restrictions.

## References

[1] Weidinger S. and Novak N. , Atopic Dermatitis, Lancet. (2016) 387, no. 10023, 1109–1122, 10.1016/s0140-6736(15)00149-x, 2-s2.0-84961201650. 26377142

[2] Nutten S. , Atopic Dermatitis: Global Epidemiology and Risk Factors, Annals of Nutrition & Metabolism. (2015) 66, no. Suppl. 1, 8–16, 10.1159/000370220, 2-s2.0-84929657504. 25925336

[3] Williams H. , Stewart A. , von Mutius E. , Cookson W. , and Anderson H. R. , Is Eczema Really on the Increase Worldwide?, Journal of Allergy and Clinical Immunology. (2008) 121, no. 4, 947–954.e15, 10.1016/j.jaci.2007.11.004, 2-s2.0-41549144352. 18155278

[4] Bieber T. , Atopic Dermatitis, Annals of Dermatology. (2010) 22, no. 2, 125–137, 10.5021/ad.2010.22.2.125, 2-s2.0-77954694888. 20548901 PMC2883413

[5] Shin Y. H. , Hwang J. , Kwon R. et al., Global, Regional, and National Burden of Allergic Disorders and Their Risk Factors in 204 Countries and Territories, From 1990 to 2019: A Systematic Analysis for the Global Burden of Disease Study 2019, Allergy. (2023) 78, no. 8, 2232–2254, 10.1111/all.15807. 37431853 PMC10529296

[6] Peng C. , Yu N. , Ding Y. , and Shi Y. , Epidemiological Variations in Global Burden of Atopic Dermatitis: an Analysis of Trends From 1990 to 2019, Allergy. (2022) 77, no. 9, 2843–2845, 10.1111/all.15380. 35587441

[7] Rademaker M. , Jarrett P. , Murrell D. F. et al., Cross-Sectional Burden-of-Illness Study in Atopic Dermatitis (MEASURE-AD) in Australia and New Zealand Reveals Impacts on Well-Being, Australasian Journal of Dermatology. (2024) 65, no. 6, 10.1111/ajd.14308. 38773888

[8] Weidinger S. , Beck L. A. , Bieber T. , Kabashima K. , and Irvine A. D. , Atopic Dermatitis, Nature Reviews Disease Primers. (2018) Nature Publishing Group. 10.1038/s41572-018-0001-z 29930242

[9] Wollenberg A. , Barbarot S. , Bieber T. et al., Consensus-Based European Guidelines for Treatment of Atopic Eczema (Atopic Dermatitis) in Adults and Children: Part I, Journal of the European Academy of Dermatology and Venereology. (2018) 32, no. 5, 657–682, 10.1111/jdv.14891, 2-s2.0-85045725596. 29676534

[10] Nygaard U. , Deleuran M. , and Vestergaard C. , Emerging Treatment Options in Atopic Dermatitis: Topical Therapies, Dermatology. (2018) 233, no. 5, 333–343, 10.1159/000484407, 2-s2.0-85037371907. 29216643

[11] Siegfried E. C. , Jaworski J. C. , Kaiser J. D. , and Hebert A. A. , Systematic Review of Published Trials: Long-Term Safety of Topical Corticosteroids and Topical Calcineurin Inhibitors in Pediatric Patients With Atopic Dermatitis, BMC Pediatrics. (2016) 16, no. 1, 10.1186/s12887-016-0607-9, 2-s2.0-84975261050. PMC4895880 27267134

[12] Hengge U. R. , Ruzicka T. , Schwartz R. A. , and Cork M. J. , Adverse Effects of Topical Glucocorticosteroids, Journal of the American Academy of Dermatology. (2006) 54, no. 1, 1–15, 10.1016/j.jaad.2005.01.010, 2-s2.0-29244466111. 16384751

[13] Aubert-Wastiaux H. , Moret L. , Le Rhun A. et al., Topical Corticosteroid Phobia in Atopic Dermatitis: A Study of Its Nature, Origins and Frequency, British Journal of Dermatology. (2011) 165, no. 4, 808–814. 21671892 10.1111/j.1365-2133.2011.10449.x

[14] Li A. W. , Yin E. S. , and Antaya R. J. , Topical Corticosteroid Phobia in Atopic Dermatitis: A Systematic Review, JAMA Dermatology. (2017) 153, no. 10, 1036–1042, 10.1001/jamadermatol.2017.2437, 2-s2.0-85031321704. 28724128

[15] Gerner T. , Haugaard J. H. , Vestergaard C. et al., Healthcare Utilization in Danish Children with Atopic Dermatitis and Parental Topical Corticosteroid Phobia, Pediatric Allergy & Immunology. (2021) 32, no. 2, 331–341, 10.1111/pai.13394. 33047404

[16] Saito-Abe M. , Futamura M. , Yamamoto-Hanada K. , Yang L. , Suzuki K. , and Ohya Y. , Topical Corticosteroid Phobia Among Caretakers of Children With Atopic Dermatitis: A Cross-Sectional Study Using TOPICOP in Japan, Pediatr Dermatol. (2019) 36, no. 3, 311–316. 30882946 10.1111/pde.13784

[17] Gomes T. F. , Kieselova K. , Guiote V. , Henrique M. , and Santiago F. , A Low Level of Health Literacy Is a Predictor of Corticophobia in Atopic Dermatitis, Anais Brasileiros de Dermatologia. (2022) 97, no. 6, 704–709, 10.1016/j.abd.2021.11.007. 36057460 PMC9582876

[18] Tangthanapalakul A. , Chantawarangul K. , Wananukul S. , Tempark T. , and Chatproedprai S. , Topical Corticosteroid Phobia in Adolescents With Eczema and Caregivers of Children and Adolescents With Eczema: A Cross-Sectional Survey, Pediatric Dermatology. (2023) 40, no. 1, 135–138, 10.1111/pde.15183. 36400426

[19] Hon K. L. , Leong K. F. , Leung T. N. , and Leung A. K. , Dismissing the Fallacies of Childhood Eczema Management: Case Scenarios and an Overview of Best Practices, Drugs In Context. (2018) 7, 212547–12, 10.7573/dic.212547, 2-s2.0-85060123020. 30532792 PMC6281040

[20] Lundborg M. , Holm J. O. H. , Sandvik L. et al., Multidisciplinary Educational Programme for Caregivers of Children With Atopic Dermatitis-in South East Norway: An Observational Study, BMC Dermatology. (2020) 20, no. 1, 10.1186/s12895-020-00119-6. PMC7727126 33298043

[21] Moret L. , Anthoine E. , Aubert-Wastiaux H. et al., TOPICOP©: A New Scale Evaluating Topical Corticosteroid Phobia Among Atopic Dermatitis Outpatients and their Parents, PLoS One. (2013) 8, no. 10, 10.1371/journal.pone.0076493, 2-s2.0-84885735578. PMC3797828 24146878

[22] Zorko M. S. , Benko M. , Rakuša M. , and Zdravković T. P. , Evaluation of Corticophobia in Patients With Atopic Dermatitis and Psoriasis Using the TOPICOP© Score, Acta Dermatovenerologica Alpina Pannonica et Adriatica. (2023) 32, no. 4, 135–139. 38126095

[23] European Task Force on Atopic Dermatitis , Clinical and Laboratory Investigations Severity Scoring of Atopic Dermatitis: The SCORAD Index Consensus Report of the European Task Force on Atopic Dermatitis, 1993, http://karger.com/drm/article-pdf/186/1/23/2637778/000247298.pdf. 10.1159/000247298 8435513

[24] Finlay A. Y. and Khan G. K. , Dermatology Life Quality Index (DLQI): A Simple Practical Measure for Routine Clinical Use, Clinical and Experimental Dermatology. (1994) 19, no. 3, 210–216, 10.1111/j.1365-2230.1994.tb01167.x, 2-s2.0-0028332995. 8033378

[25] Basra M. K. A. , Sue-Ho R. , and Finlay A. Y. , The Family Dermatology Life Quality Index: Measuring the Secondary Impact of Skin Disease, British Journal of Dermatology. (2007) 156, no. 3, 528–538, 10.1111/j.1365-2133.2006.07617.x, 2-s2.0-33846988561. 17300244

[26] Contento M. , Cline A. , and Russo M. , Steroid Phobia: A Review of Prevalence, Risk Factors, and Interventions, American Journal of Clinical Dermatology. (2021) 22, no. 6, 837–851, 10.1007/s40257-021-00623-6. 34287768

[27] Albogami M. F. , Aljomaie M. S. , Almarri S. S. et al., Topical Corticosteroid Phobia Among Parents of Children With Atopic Dermatitis (Eczema): A Cross-Sectional Study, Patient Preference and Adherence. (2023) 17, 2761–2772, 10.2147/ppa.s431719. 37936715 PMC10627058

[28] Mueller S. M. , Itin P. , Vogt D. R. et al., Assessment of Corticophobia as an Indicator of Non-Adherence to Topical Corticosteroids: A Pilot Study, Journal of Dermatological Treatment. (2017) 28, no. 2, 104–111, 10.1080/09546634.2016.1201189, 2-s2.0-84978148225. 27396480

[29] Krejci-Manwaring J. , Tusa M. G. , Carroll C. et al., Stealth Monitoring of Adherence to Topical Medication: Adherence Is Very Poor in Children With Atopic Dermatitis, Journal of the American Academy of Dermatology. (2007) 56, no. 2, 211–216, 10.1016/j.jaad.2006.05.073, 2-s2.0-33846085514. 17224366

[30] Smith S. D. , Hong E. , Fearns S. , Blaszczynski A. , and Fischer G. , Corticosteroid Phobia and Other Confounders in the Treatment of Childhood Atopic Dermatitis Explored Using Parent Focus Groups, Australasian Journal of Dermatology. (2010) 51, no. 3, 168–174, 10.1111/j.1440-0960.2010.00636.x, 2-s2.0-77955286959. 20695854

[31] Lee J. Y. , Her Y. , Kim C. W. , and Kim S. S. , Topical Corticosteroid Phobia Among Parents of Children With Atopic Eczema in Korea, Annals of Dermatology. (2015) 27, no. 5, 499–506, 10.5021/ad.2015.27.5.499, 2-s2.0-84943793724. 26512163 PMC4622883

[32] Herzum A. , Occella C. , Gariazzo L. , Pastorino C. , and Viglizzo G. , Corticophobia Among Parents of Children With Atopic Dermatitis: Assessing Major and Minor Risk Factors for High TOPICOP Scores, Journal of Clinical Medicine. (2023) 12, no. 21, 10.3390/jcm12216813. PMC10650526 37959278

[33] Lambrechts L. , Gilissen L. , and Morren M. A. , Topical Corticosteroid Phobia Among Healthcare Professionals Using the TOPICOP Score, Acta Dermato-Venereologica. (2019) 99, no. 11, 1004–1008, 10.2340/00015555-3220, 2-s2.0-85073464281. 31099401

[34] Finnegan P. , Murphy M. , and O’connor C. , Corticophobia: A Review on Online Misinformation Related to Topical Steroids, Clinical and Experimental Dermatology. (2023) 48, no. 2, 112–115, 10.1093/ced/llac019. 36730502

[35] Stalder J. F. , Aubert H. , Anthoine E. et al., Topical Corticosteroid Phobia in Atopic Dermatitis: International Feasibility Study of the TOPICOP Score, Allergy: European Journal of Allergy and Clinical Immunology. (2017) 72, no. 11, 1713–1719, 10.1111/all.13189, 2-s2.0-85019941555. 28439896

[36] Brunner C. , Schlüer A. B. , Znoj H. et al., Video-Based Education With Storytelling Reduces Parents’ Fear of Topical Corticosteroid Use in Children With Atopic Dermatitis: A Randomized Controlled Trial (The Eduderm Study Part II), Advances in Skin & Wound Care. (2023) 36, no. 8, 414–419, 10.1097/asw.0000000000000002. 37471446

